# Gender-related responses of dioecious plant *Populus cathayana* to AMF, drought and planting pattern

**DOI:** 10.1038/s41598-020-68112-0

**Published:** 2020-07-13

**Authors:** Zhen Li, Na Wu, Ting Liu, Ming Tang, Hui Chen

**Affiliations:** 1 State Key Laboratory of Conservation and Utilization of Subtropical Agro-Bioresources, Lingnan Guangdong Laboratory of Modern Agriculture, Guangdong Key Laboratory for Innovative Ddevelopment and Utilization of Forest Plant Germplasm, College of Forestry and Landscape Architecture, South China Agricultural University, Guangzhou, 510642 China; 2 School of Life Sciences, Shanxi Datong University, Datong, 037009 China; 3 College of Biology and Agriculture, Zunyi Normal College, Zunyi, 563000 China

**Keywords:** Plant physiology, Fungi

## Abstract

In our previous studies, we detected drought, gender and arbuscular mycorrhizal (AM) inoculation effects on dioecious plant. Based on this, we investigated the intra- and inter-sexual competition between male and female plants. Dioecious plant *Populus cathayana* was used and we set 3 factors in this experiment: (1) AM inoculation/non-inoculation; (2) well-watered/water-stressed; (3) single-gender pattern (only 4 males or 4 females)/mixed-gender pattern (2 males and 2 females). Growth (stem length, ground diameter, SPAD, mean leaf area, biomass accumulation) and nutrition (C, N, P, K, Ca and Mg) distribution of male and female seedlings were determined. *Results* Drought significantly limited plant growth and nutrition accumulation, especially in female plants; AM formation alleviated this negative effect, especially in male plants. However, the gender effect was complicated. A mixed-gender planting pattern relieved intra- competition in terms of the growth and nutrient accumulation of both genders and even alleviated the negative effects caused by drought. In the mixed-gender pattern, the differences of C, N, P, K and Ca contents between male and female plants with AM inoculation was smaller than those without AM inoculation, which indicated a potential role for AM fungi in nutrient transport. Males had a stronger physiological response to limited water availability, and more advantages from AM formation than females. Mixed-gender planting relieved the existence of intra- sexual competition of dioecious plants, and AM symbiosis alleviated the differences between genders.

## Introduction

Forests cover 30% of the world’s land surface and are relied on by human societies. However, land use by our expanding human population and economic decisions are rapidly and directly transforming forested ecosystems^1^. With increasingly higher global temperature, which is now widely acknowledged because of increasing emissions of greenhouse gases, significant drying is occurring widely around the world^2,3^. The impacts of climate change have both positive and negative sides. However, increases in water use efficiency, growth from higher CO_2_ levels, and the growing season suggest positive effects of future climate change on forests. The changing climate induces stress and changes the dynamics of forest insects and pathogens, which reduce tree growth and increase mortality^4,5^.

Afforestation is a well-accepted method for ecological restoration in arid areas^6^, and poplar is a widely cultivated forest tree with high economic value, especially in the energy and papermaking industries^7,8^. *Populus cathayana,* a typical dioecious plant, is an ecologically important species in Qinghai Province, China, a severely ecologically degraded area. Despite decades of studies within the fields of forestry, plant pathology and entomology, the fundamental mechanisms of tree survival and mortality during drought remain poorly understood^9–11^.

Most terrestrial plants can establish symbiosis with arbuscular mycorrhizal (AM) fungi, which is widespread, and has been suggested to enhance the inherent ability of the plant to tolerate stresses and to alleviate stress symptoms^12,13^. Mycorrhizal plants could get better tolerance to kinds of stress owing to enhanced transportation of water and nutrients (especially phosphorus) by AM symbiosis, which is essential for plant growth and development^13^. Plants that form AM symbiosis have been shown to be markedly more resistant to drought^14^, pathogens^15^, heavy metals^16^ and herbivorous insects^17^. Under most harsh environmental conditions, AM fungal symbioses are known as bioenhancers, and many field and pot experiments have produced similar results^18,19^.

Male and female plants of dioecious plant species differ in morphology, physiology and biochemistry, especially under various environmental stresses. Generally, the gender difference is determined by different reproductive costs: female plants must provide more to reproduce^20^. Allelopathy existed between plant species and individuals, even between microorganisms and between plants and microorganisms^21,22^. However, the differences in allelopathy between the two genders of dioecious plants have not been well studied. In addition, in the wild environment, a plant rarely grows by itself, and the influence from neighbouring plants, especially those of the same species, must be considered. AM fungi form symbioses with different plants at the same time, which also facilitates communication channels between the plants, which are known as common mycorrhizal networks^23^. Through this network, plants transport signals, even water and nutrition, which helps plants overcome various kinds of stresses^24,25^. We hypothesize that there may be some special communication between genders, and this communication may be facilitated by AM fungi. Therefore, we designed this experiment to investigate the potential role of AM fungi in plant communication and to identify potential effective ways to alleviate the gender imbalance.

## Results

### Comparison between water regimes in weight lost of pots every day

In the early 30 days, the pots lost about 1,600 g in weight every day. The field capacity of pots in drought treatments spent 5 days changing from 85–90 to 25–30% before the drought treatment. During the course of drought period, the average weights lost every day in well-watered treatment (WW)and water-stressed condition (WS)were about 1,600 g and 1,300 g, respectively, and showed slight increases with time.

### Inoculation status

The inoculation rates are shown in Supplementary Fig. 1. All inoculation rates from the different inoculated treatment groups were over 80%. Among the same gender, inoculation rates of plants from WW were higher than those from WS. Meanwhile, no significant effects were detected between genders and planting patterns (mixed-gender or single-gender).

**Figure 1:**
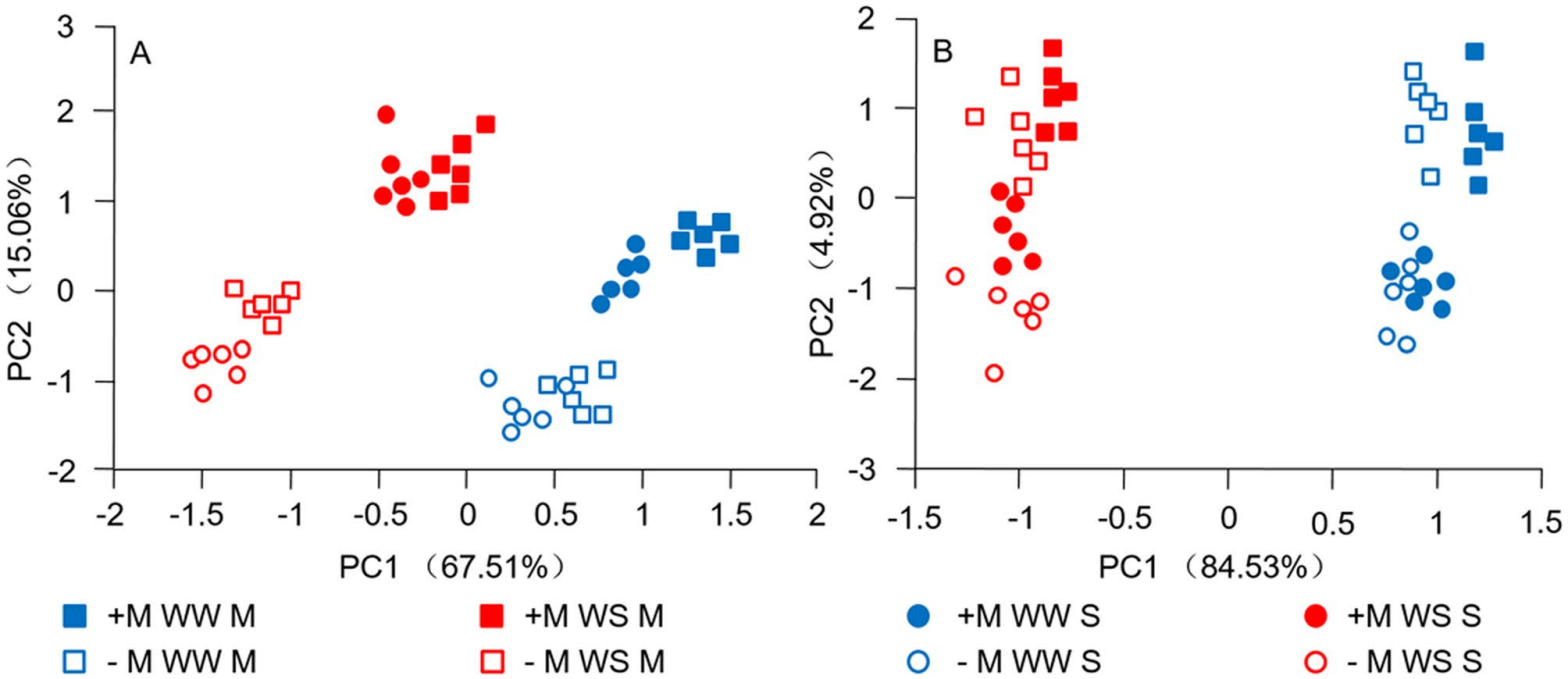
PCA results. **a** and **b** showed PCA results of male and female plants respectively; + M: inoculated treatment; -M: non-inoculated treatment; S: single-gender planting; M: mixed-gender planting.

### Growth indexes

Before harvest, 6 plants from each treatment were randomly selected for stem length, ground diameter, soil and plant analyser development (SPAD) value and leaf area (LA) measurements (Supplementary Fig. 2). Different watering treatments had the same impact on both genders: stem length, ground diameter, SPAD and LA values of both genders were significantly limited by drought, and this negative effect of drought was greater in females. Meanwhile, the effects of different inoculation treatments differed between genders: among male plants, AM inoculation notably improved stem length and LA, especially under drought conditions; among female plants, inoculation treatment had no significant impacts. Meanwhile, the LA of female plants was significantly greater than that of male plants. However, male plants performed much better than female plants in stem length, ground diameter and SPAD, which indicated better drought resistance of male plants than female plants.

**Figure 2:**
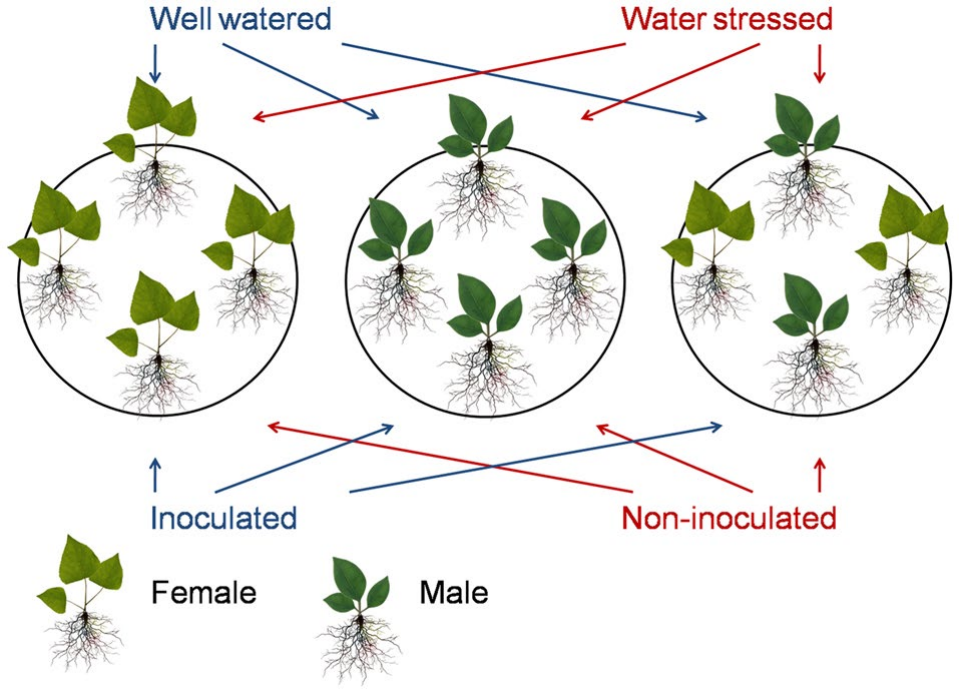
Experimental design.

Compared with single-gender planting, mixed-gender planting improved the stem length, ground diameter and SPAD value of both genders. In addition, mixed-gender planting improved the LA of female plants. Analysis of variances (ANOVA) results showed that all growth indexes were significantly affected by inoculation, drought and planting pattern treatments, and they were also significantly affected by gender (except SPAD), the interactions of gender × inoculation, gender × drought, and gender × planting pattern.

Supplementary Fig. 3 shows the changes in stem length and ground diameter of both genders during 50 days of water treatment. Initially, no obvious differences were detected between treatments. However, a few days later, among plants under WS, stem length and ground diameter increased slowly, but the growth rate improved greatly after 20 days. Meanwhile, under WW, plants grew stably at the beginning, but the growth rate decreased after 30 days. Apart from water treatment, differences in growth rates of stem length and ground diameter between genders and planting pattern treatments appeared after 10 days.

### Biomass

The biomass results are shown in Supplementary Fig. 4. The dry weight of shoot (DWS), dry weight of root (DWR), total dry weight (TDW) and root/shoot ratio (RSR) under different water, inoculation and planting pattern treatments differed. Drought limited DWS, DWR and TDW significantly in both genders. Drought treatment significantly increased RSR, especially in female plants. Drought affected female plants most significantly. However, in female plants under the same water treatment, no significant difference was detected between inoculation treatments or planting pattern treatments. Among male plants, under the same water and planting pattern treatments, inoculation improved the biomass accumulation of plant parts. However, mixed-gender planting had different effects on male and female plants: The biomass of male plants from the mixed-gender planting pattern accumulated more than that from male only plantings. However, among female plants, this effect existed only under WW conditions. At the same time, the mixed-gender planting pattern decreased RSR, especially among male plants. However, under the same water treatment, differences between other treatments were not significant.

Two-way ANOVA results showed that all of the treatments had significant effects on male DWS, DWR and TDW; only water treatment had an apparent impact on female DWS, DWR, TDW and RSR. Three-way ANOVA showed that biomass and RSR were significantly affected by gender and the interactions between gender × inoculation, gender × drought, and gender × planting pattern.

### C, N and P contents in male and female leaves and roots

The distribution of C, N and P is shown in Supplementary Fig. 5. The values of C and N contents in the roots were close to those in the leaves, while P was distributed more in the leaves. Compared with plants under WW conditions, plants under WS conditions had significantly less C, N and P accumulation of whole plant. This depressor effect was greater on female plants than on males.

To some extent, AM inoculation alleviated this negative effect on male plants, but its effect on female plants was complicated. Among male seedlings, inoculation treatment significantly improved the C of both the root and leaf and the N and P contents of roots, while significant improvement in the N and P contents of leaves existed only under WW conditions. Among female seedlings, inoculation treatment clearly improved the C concentration of leaves under WW conditions and the N and P concentrations of roots under WS conditions. However, under WS conditions, AM inoculation decreased the C concentrations of both the leaves and roots of female plants.

Apart from the effects of the water and inoculation treatments, different planting patterns also affected plant performance. As shown, compared with plants with a single-gender planting pattern, plants with a mixed-gender planting pattern showed higher C, N and P accumulation. In this study, we found that the total N concentration of both root and shoot of female plants was higher than that of male plants. Meanwhile, C, N and P accumulated slightly more in plants with a mixed-gender planting pattern than in plants with a single-gender planting pattern. We also detected that under a mixed-gender planting pattern, the gaps between male and female plants under the inoculated treatment were smaller than those under non-inoculated treatment.

Two-way ANOVA results showed that among male plants, inoculation, water and planting pattern treatments had significant effects on C (except leaf), N and P contents of both the root and leaf (Supplementary Table 1). Meanwhile, the interactions of inoculation treatment × water treatment significantly affected the C and N contents of the leaf and C, N and P contents of the root. Among female plants, inoculation treatment had no significant effects on the C, N and P contents of leaves but had significant effects on the concentration of these elements in the roots. Moreover, the interaction of inoculation treatment × water treatment significantly affected the C, N and P contents of both roots and leaves. The interaction of the inoculation × planting pattern also clearly affected the N and P accumulation of both roots and leaves. Three-way ANOVA results showed that, except for the P content of the leaf, the C, N and P contents from other plant parts were significantly affected by gender. In addition, contents of all 3 elements were significantly affected by the interactions of gender × inoculation, gender × drought, gender × planting pattern and gender × inoculation × drought. The N and P contents of both roots and leaves were also significantly affected by the interaction of gender × inoculation × planting pattern.

### K, Ca, Mg contents in male and female leaves and roots

The distribution of K, Ca and Mg contents between different treatments is shown in Supplementary Fig. 6. K was distributed more in roots than in leaves, but Ca and Mg showed opposite results. In contrast to C, N and P, drought treatment significantly improved the K and Ca concentrations of roots and leaves. However, drought significantly limited Mg accumulation, especially among female plants. In addition, we found that the range of K was clearly smaller than that of Ca and Mg. Compared with the effect of inoculation on C, N and P accumulation, this positive effect on K and Ca was less pronounced. A significant increase in Ca concentrations of only male roots and leaves was detected.

Similar to the results for C, N and P concentrations, compared with plants in a single-gender planting pattern, plants in mixed-gender planting patterns showed better performance in K, Ca and Mg accumulation, especially in Ca contents. In addition, among K and Ca, the presence of AM fungi often alleviated the gap between genders in the mixed-gender planting pattern. However, the distribution of Mg showed a distinct pattern: AM inoculation increased the gender difference.

Two-way ANOVA results showed that among male plants, K, Ca and Mg concentrations were significantly affected by inoculation, water and planting pattern treatments and were also significantly impacted by the interactions of inoculation × drought, inoculation × planting pattern and the interaction of 3 factors (Supplementary Table 1). The K content of the roots was clearly affected by the inoculation and water treatments, and the Ca and Mg contents in the roots were significantly impacted by all 3 factors. Among female seedlings, inoculation, water and planting pattern treatments had significant effects on K and Ca concentrations of both the root and leaf, and K and Ca contents of the leaf, K and Mg contents of the root were remarkably affected by the interaction of inoculation × drought. Three-way ANOVA results indicated that the K, Ca and Mg contents of leaves were significantly affected by gender and the interactions of inoculation × gender, drought × gender, and planting pattern × gender. Meanwhile, the effects of gender and interactions of inoculation × gender and drought × gender were significant for the K, Ca and Mg contents of roots.

### PCA results

To investigate whether male and female plants showed different responses in growth, biomass accumulation and nutritional distribution to drought, AM inoculation and planting pattern treatments, PCA was performed using the experimental data above. Among male plants, PC1 and PC2 accounted for 67.51% and 15.06% of the variance, respectively (Fig. 1a). PC1 tended to separate the drought and planting pattern effects at the same time. DWS, TDW, stem length, ground diameter, and C, P contents of roots and leaves were the key contributors to PC1 (Supplementary Table 2). PC2 tended to uncouple the difference in AM inoculation. Among female plants, PC1 and PC 2 accounted for 84.53% and 4.92% of the variance, respectively (Fig. 1b). PC1 tended to separate the drought effects, and PC2 tended to uncouple the differences of AM inoculation and planting pattern at same time. DWS, DWR, TDW, stem length, ground diameter, N content of the root, and N, P, Mg contents of the leaf were the key contributors to PC1. These results showed that among female seedlings, the main effects were from the water treatment only. Male plants showed a stronger physiological response to limited water availability than female plants, and AM inoculation had a positive effect on the drought tolerance of both genders, especially on that of males. In addition, compared with plants in a single-gender planting pattern, male and female plants in a mixed-gender planting pattern performed better.

## Discussion

In our previous studies^21,22^, gender differences in physiological responses to drought and AM inoculation were investigated. Therefore, we aimed to discuss the potential effects between genders and their interactions with AM fungi under both WW and WS conditions.

Among a factors in this study, drought treatment affected greater than the other two. Drought limited root AM inoculation rates of both genders, which agrees with the results of most studies^21,22^. Water is one of the key factors in photosynthesis, and limited water availability severely restricted plant growth. Generally, limited water availability results in a decrease in stem length, ground diameter, SPAD values, LA and biomass accumulation, which was in line with our results. Moreover, decreased SPAD values resulted in limited photosynthesis. In this study, at the beginning of the water treatment, no obvious differences were detected between treatments. However, after 10 to 20 days, among plants under drought treatments, stem length and ground diameter increased slightly, but the growth rate improved greatly after 20 days. This phenomenon was caused by the fact that, during this period, plants under drought conditions had to adjust their growth to the new water conditions. However, plants under well-watered conditions grew stably at the beginning, but the growth rate decreased after 30 days. After 40 days, plants under both conditions grew slowly. We hypothesized that two reasons may cause this: the growth characteristics of *P. cathayana* or the limitations of the pots.

Drought limited plant growth and biomass accumulation of each plant part of both genders. However, in keeping with previous studies, drought significantly increased the RSR, which was due to the limitation of growth by the water deficit^21^. Roots acted as the main tissues for water uptake, receiving less influence from drought. In addition, drought limited C, N and P absorption, because the water deficit of soil reduced the water with which soil elements were absorbed. However, in contrast to C, N and P, drought treatment significantly improved the K and Ca concentrations of roots and leaves. This result was due to their important roles in osmotic adjustment, and their higher concentration alleviated the negative effects of drought.

The presence of AM symbiosis improved the tolerance of plants to most environmental stress, of which drought tolerance was most reported^26,27^. Similar to previous studies, we found that AM inoculation increased the drought tolerance of *P. cathayana* in terms of growth, biomass and nutrient accumulation^21,22^. AM symbiosis not only improved the growth status of plants but also promoted the secretion of roots and hyphae, which indirectly improved the soil condition and water status^24,25^. In other words, AM formation improved the water status of plants in many ways.

AM formation improved the absorptive capacity of nutrients in plants, which made AM fungi essential for plants living in poor environments. Improvement in P absorption was reported most often. P transporters in AM fungi helped plants absorb inorganic P^28^. Research has shown that P absorbed by AM fungi could be 5 times that absorbed by plants only^29^. Consistent with this study, AM formation increased P accumulation in *P. cathayana*. In addition, AM symbiosis improved the absorption of various types of nutrients, including heavy metals, such as N^30^, K^31^, Zn^32^, Cu, Fe and Mn^33^. We also found that the presence of AM fungi had a positive effect on the nutrient accumulation of *P. cathayana* males and females.

In our previous studies^21,22^, *P. cathayana* male and female seedlings differed in physiological responses to AM formation, which agrees with the results of this experiment. Among male plants, AM inoculation increased plant growth, especially under WS conditions. Among female plants, AM formation resulted in less improvement in growth, while a significant effect was detected only in stem length. The improvement was caused by the direct and indirect improvements of the plant water status by AM formation. Besides, different gender characteristics and function counted: the gender difference is determined by different reproductive costs: female plants must provide more to reproduce^20^^.^ That why females grew better under WW conditions, but much worse under WS condition than males in terms of growth indexes, which also indicated that female plants were potentially more sensitive to environmental stress, even with AM formation^34,35^.

In nature, males and females of dioecious plants differed on several levels, such as morphology, physiology and biochemistry. In recent years, with the deteriorating environment, the gender ratios of many dioecious plants have become imbalanced. Chen et al. found that male *P*. *cathayana* showed higher photosynthesis and less cellular injury than females under high manganese condition^34^. Han et al. suggested that *P*. *cathayana* showed gender differences under drought stress, while reciprocal grafting could alleviate negative effects by gender^35^. Most reports have shown that, in the face of environmental stresses, male plants perform better than female ones not only under abiotic stresses, such as drought, salt and UV stress, but also under biotic stresses^21,22,34,35^.

In this study, there was no significant difference in AM inoculation rates between genders under different treatments, which was in line with our previous studies^21,22^. We found that male and female plants showed different responses to different treatments, especially drought. Under drought conditions, male plants performed much better, and the AM formation was much higher for males, which was also consistent with previous studies^8,35^.

However, the mixed-gender planting pattern significantly increased the LA of female plants, which was an interesting result. We hypothesized that there may be one or many elements that were essential in LA growth, and a mixed-gender planting pattern alleviated competition, resulting in a larger LA of female plants. For example, we detected this effect under normal water conditions, the Mg concentration of female plants was much higher than that of male plants. Moreover, the distribution of Mg showed a distinct pattern compare to other elements measured: AM inoculation increased the gender difference of Mg. We hypothesized that the characteristic Mg preferences of male and female plants may have caused this result. More research is needed to uncover this phenomenon.

The effects of different planting patterns were the most notable highlight in this experiment. We considered that in the wild environment, the direct effect of losing the gender balance was reduced reproductive efficiency. However, in previous studies, interactions between male and female plants were ignored. These interactions did not remain the same at all times. They would generally change with the environment. As constrained resources such as water and soil elements shrink, the relationship between plants changed from negative to positive^36^. AM inoculation could change the relationship between plants^37,38^, and different AM fungal strains showed different effects^39^.

We found that male and female plants in the mixed-gender planting pattern performed slightly better than those in the single-gender planting pattern. We hypothesized that due to different demands for soil elements among different genders, when male and female plants were planted together, less competition occurred. In this case, both male and female plants grew better and experienced more nutrient uptake. Plants of the same gender competed for the same elements, and the mixed-gender planting pattern relieved this competition.For example, in this study, we found that the total N concentration of both root and shoot of female plants was higher than that of male plants, which indicates the potential for more demand for N in female plants than in male plants. Moreover, the results of the nutrient distribution supported our explanation: female plants showed a preference for N and Mg. In addition, we hypothesized that there may be some special communication between genders that influenced the nutrient distribution. This communication pathway may be via AM fungi in this experiment. AM fungi formed symbioses with different plants at the same time, which also facilitated communication channels between plants, which are called common mycorrhizal networks^23^. Through this network, plants transported signals, even water and nutrients, which helped plants overcome various kinds of stresses^24,25^.

Apart from improvement in growth, the mixed-gender plating pattern increased nutrient accumulation to a certain extent. Furthermore, we found that in the mixed-gender planting pattern, the differences in C, N, P, K and Ca concentrations between inoculated male and female plants was smaller than that between non-inoculated male and female plants. The presence of AM symbiosis formed networks between plants, and nutritional transport alleviated the gap between genders. However, the mixed-gender planting pattern increased the gap of Mg between male and female seedlings. This may be caused by the special characteristics of Mg and the preference of females for Mg. We found that under WW conditions, female plants had much higher Mg concentrations than males. More research is needed in the future to investigate all of the mechanisms involved in these experimental effective ways to aid *P. cathayana* survival and reproduction, but proper utilization of AM fungi may be one potential solution.

## Methods

### Plant and soil treatment

We obtained permission and help from the owner of a poplar nursery (101° 31′ 48" E, 37° 2′ 24" N) in Sining, Qinghai Province, China, to conduct the study on this site. From this nursery, we collected 96 different 1-year-old male and female *P*. *cathayana* seedlings (48 of each gender) that were sampled from 12 populations (8 adult trees of the same age per population). The seedlings were then cut into cuttings, 18 cm in length and 1.2 cm in diameter. The cuttings were disinfected with 70% ethanol (v/v) for 15 s and rinsed 3 times with sterile deionized water^24^.

From a poplar nursey in Yangling, Shaanxi Province, China, we collected topsoil (5–20 cm) and sieved it through a 2-mm sieve, before mixing it with fine sand (v:v = 1:1). The mixture was then autoclaved under pressure (0.11 MPa) at 121 °C for 2 h as the matrix in this experiment. The soil physicochemical properties were as follows: available N, 37.50 mg/kg; available P, 12.34 mg/kg; available K, 133.24 g/kg; and organic matter, 18.76 g/kg; pH, 7.6 (measured in soil: water using a 1:5 ratio).

### AM inoculum

The AM inoculum in this study was from *Rhizophagus intraradices* JJ291 (BEG accession 158 at the International Bank for the Glomeromycota; https://www.hent.ac.uk/bio/beg/) and consisted of spores (spore density was approximately 50/g inoculant), mycelia, root fragments and soil.

### Experimental design

To investigate intra- and inter-sexual competition, we planted 4 seedlings in one pot. As shown in Fig. 2, the experimental layout included 3 factors: an inoculation treatment (with and without *R. intraradices* inoculation), water treatment [well-watered (WW) or water-stressed (WS)] and planting pattern treatment [single-gender (4 seedlings of same gender) or mixed-gender (2 male and 2 female seedlings)]. WW and WS treatments utilized 85–90% and 25–30% of the soil field capacity, respectively 24 (Li et al. 2015). Four cuttings were planted in one pot, and different planting patterns included the following: 4 male cuttings, 4 female cuttings and mixed cuttings (with 2 male and 2 female cuttings). Half of the pots were mixed-gender plantings, and 1/4 of the pots were male only or female only to maintain the same number of repetitions between different planting patterns.

In this experiment, to contain 4 seedlings, plastic pots (35 cm in height, 25 cm in root diameter and 40 cm in aperture diameter) filled with 30 kg preconditioned soil matrix were used. All of the pots were kept in a greenhouse at 25–30 °C with 12 h of light per day. Pots with AM inoculation treatment were inoculated with AM inoculum (20 g/plant), and the remaining pots were inoculated with 20 g of autoclaved inoculum with 10 ml of inoculum washing solution that had been filtered through a 1-μm nylon mesh to remove the live inoculum. After 30 days, among each gender, half of the inoculated pots were subjected to drought treatment and were left un-watered until the soil reached 25–30% of field capacity. The rest of the pots were kept at 85–90% of field capacity. Finally, 6 replicates were designed for each treatment. Throughout the experiment, all pots were weighed and watered every day at 16:00 h to maintain the experimental soil field capacity. The pots were harvested after 50 days.

### Growth measurement

Throughout the experiment, stem length and ground diameter were measured by tape and Vernier calipers every 5 days. At the end of the experiment, leaf area (LA) was measured using coordinate paper. The chlorophyll content [soil and plant analyser development (SPAD) value] was measured with a chlorophyll metre (SPAD-502 Plus, Konica-Minolta Holdings, Inc., Osaka, Japan). At the end of the experiment, all seedlings were divided into aboveground and belowground parts. Each part was dried at 70 °C to a constant weight and the dry weight of the shoot (DWS), dry weight of the root (DWR), and total dry weight (TDW) were determined. The ratio of DWR to DWS was calculated as the root/shoot ratio (RSR).

### Root inoculation rate measurement

Samples of the fresh roots were collected immediately after plants were harvested, gently washed, cut into 1-cm pieces, and fixed with FAA solution. 10% KOH and 0.05% trypan blue in lactophenol were used to clear and stain the root samples^40^. Root colonization was examined under the microscope and evaluated as described by Giovanetti and Mosse^41^. Data are given as the percentage of colonized root length.

### Nutrient distribution measurement

Root and leaf samples were dried at 105 °C for 30 min, and then at 70 °C to constant weight, gounded and sieved through a 0.15 mm sieve. The N and C concentrations were measured using the semi micro-Kjeldahl method^42^ and the K_2_Cr_2_O_7_ method^43^. P content was measured with the molybdenum blue spectrophotometry method^43^. K, Ca and Mg concentrations were measured using flame atomic absorption spectrophotometry (FAAS)^44,45^.

### Statistical analysis

Experimental data were subjected to two-way and three-way analysis of variances (ANOVA) using the statistical software package SPSS 17.0 (SPSS Inc., Chicago, II., USA). The means were compared by Duncan’s multiple-range tests (*p* ≤ 0.05) in two-way and three-way ANOVAs. Two-way ANOVA was performed to determine the significance of the effects of inoculation, water and planting pattern treatments, the interactions of inoculation × drought, inoculation × planting pattern, drought × planting pattern and their interactions with male and female plants. Three-way ANOVA was used to evaluate the significance of the effects of gender, the interactions of gender × inoculation, gender × drought, gender × planting pattern, and the interactions of any 3 factors and all 4 factors. Data are shown as the means ± SD. For principal component analysis (PCA), data were standardized and subsequently computed.

## Supplementary information

Supplementary file1 (DOCX 13 kb)

Supplementary file2 (DOCX 12 kb)

Supplementary file3 (TIF 117 kb)

Supplementary file4 (TIF 2322 kb)

Supplementary file5 (TIF 807 kb)

Supplementary file6 (TIF 2234 kb)

Supplementary file7 (TIF 1660 kb)

Supplementary file8 (TIF 1125 kb)

Supplementary file9 (DOCX 45 kb)

Supplementary file10 (DOCX 24 kb)
