# Supplementary material for: Gender-related responses of dioecious plant Populus cathayana to AMF, drought and planting pattern
Source: Sci Rep. 2020 Jul 13;10:11530. doi: 10.1038/s41598-020-68112-0 (PMC7359309; doi:10.1038/s41598-020-68112-0)
Supplement: Supplementary file 1 — Supplementary file1 (DOCX 13 kb) [file 41598_2020_68112_MOESM1_ESM.docx]

**Legends of Supplementary Figures**

**Supplementary Figure 1.** Inoculation rates

**Supplementary Figure 2.** Stem length (a), ground diameter (b), SPAD (c) and LA (d) of *Populus cathayana* males and females

+M: inoculated treatment; -M: non-inoculated treatment; S: single-gender planting; M: mixed-gender planting. Different letters on the error bar indicate significant difference at *p* ≤ 0.05.

**Supplementary Figure 3.** Changes in stem length and ground diameter of *Populus cathayana* males and females

a, c: single-gender planting; b, d: mixed-gender planting; M: male; F: female; +M: inoculated treatment; -M: non-inoculated treatment.

**Supplementary Figure 4.** DWS (a), DWR (b), TDW (c) and RSR (d) of *Populus cathayana* males and females

Different letters on the error bar indicate significant difference at *p* ≤ 0.05.

**Supplementary Figure 5.** C, N and P concentrations of leaf and root of *Populus cathayana* males and females

+M: inoculated treatment; -M: non-inoculated treatment; S: single-gender planting; M: mixed-gender planting. Different letters on the error bar indicate significant difference at *p* ≤ 0.05.

**Supplementary Figure 6.** K, Ca and Mg concentrations of leaf and root of *Populus cathayana* males and females

+M: inoculated treatment; -M: non-inoculated treatment; S: single-gender planting; M: mixed-gender planting. Different letters on the error bar indicate significant difference at *p* ≤ 0.05.
