# Supplementary material for: Gender-related responses of dioecious plant Populus cathayana to AMF, drought and planting pattern
Source: Sci Rep. 2020 Jul 13;10:11530. doi: 10.1038/s41598-020-68112-0 (PMC7359309; doi:10.1038/s41598-020-68112-0)
Supplement: Supplementary file 2 — Supplementary file2 (DOCX 12 kb) [file 41598_2020_68112_MOESM2_ESM.docx]

**Gender-related responses of dioecious plant *Populus cathayana* to AMF, drought and planting pattern**

Zhen Li^2,#^, Na Wu^2,#^, Ting Liu^3^, Hui Chen^1^, Ming Tang^1*^

1. State Key Laboratory of Conservation and Utilization of Subtropical Agro-Bioresources, Lingnan Guangdong Laboratory of Modern Agriculture, Guangdong Key Laboratory for Innovative Ddevelopment and Utilization of Forest Plant Germplasm, College of Forestry and Landscape Architecture, South China Agricultural University, Guangzhou, 510642, China

2. School of Life Sciences, Shanxi Datong University, Datong, 037009, China

3. College of Biology and Agriculture, Zunyi Normal College, Zunyi, 563000, China

^#^ Zhen Li and Na Wu have contributed equally to this work

^*^Corresponding author: Ming Tang

E-mail address: [tangmingyl@163.com](mailto:tangm@nwafu.edu) (Tang Ming)

Telephone: 86 13709229152
