# Supplementary figures and images for: Gender-related responses of dioecious plant Populus cathayana to AMF, drought and planting pattern
Source: Sci Rep. 2020 Jul 13;10:11530. doi: 10.1038/s41598-020-68112-0 (PMC7359309; doi:10.1038/s41598-020-68112-0)

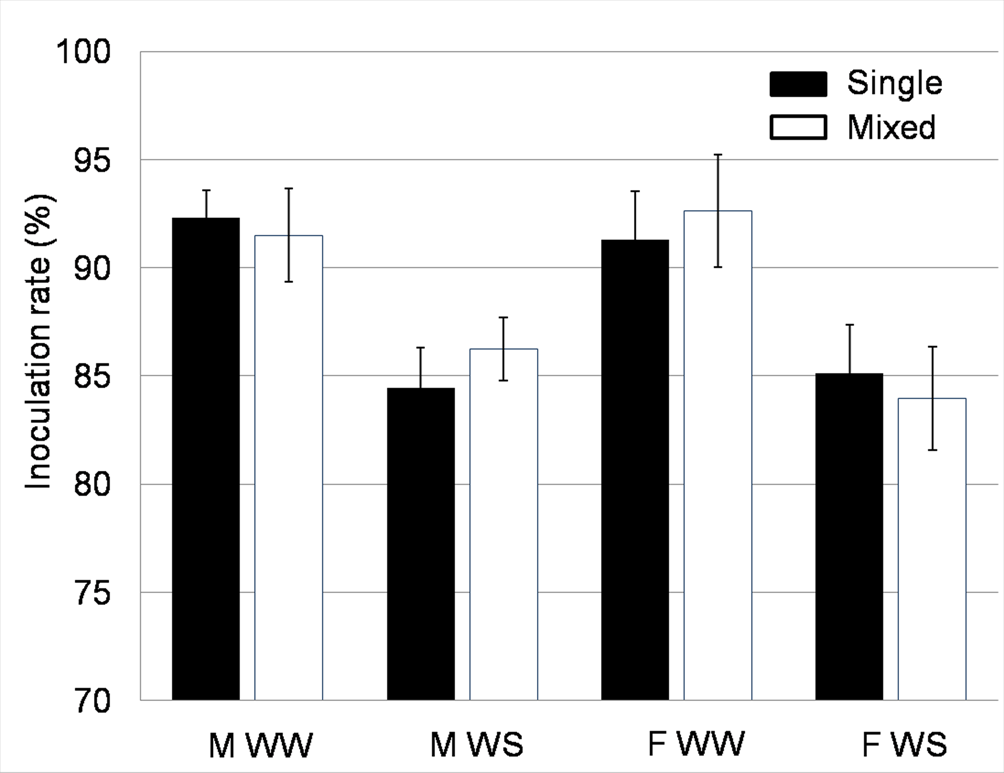

Supplement: Supplementary file 3 — Supplementary file3 (TIF 117 kb) [file 41598_2020_68112_MOESM3_ESM.tif]

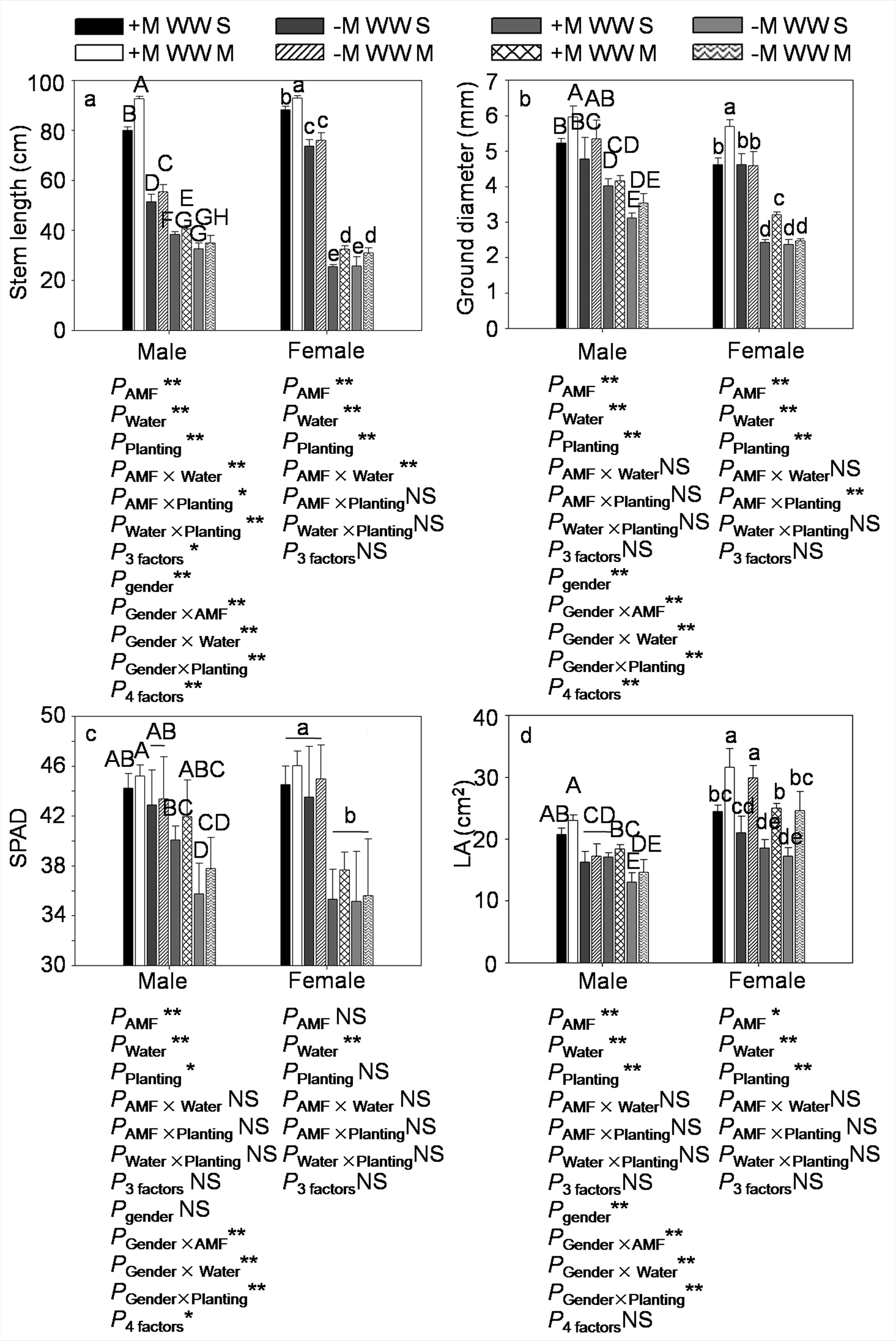

Supplement: Supplementary file 4 — Supplementary file4 (TIF 2322 kb) [file 41598_2020_68112_MOESM4_ESM.tif]

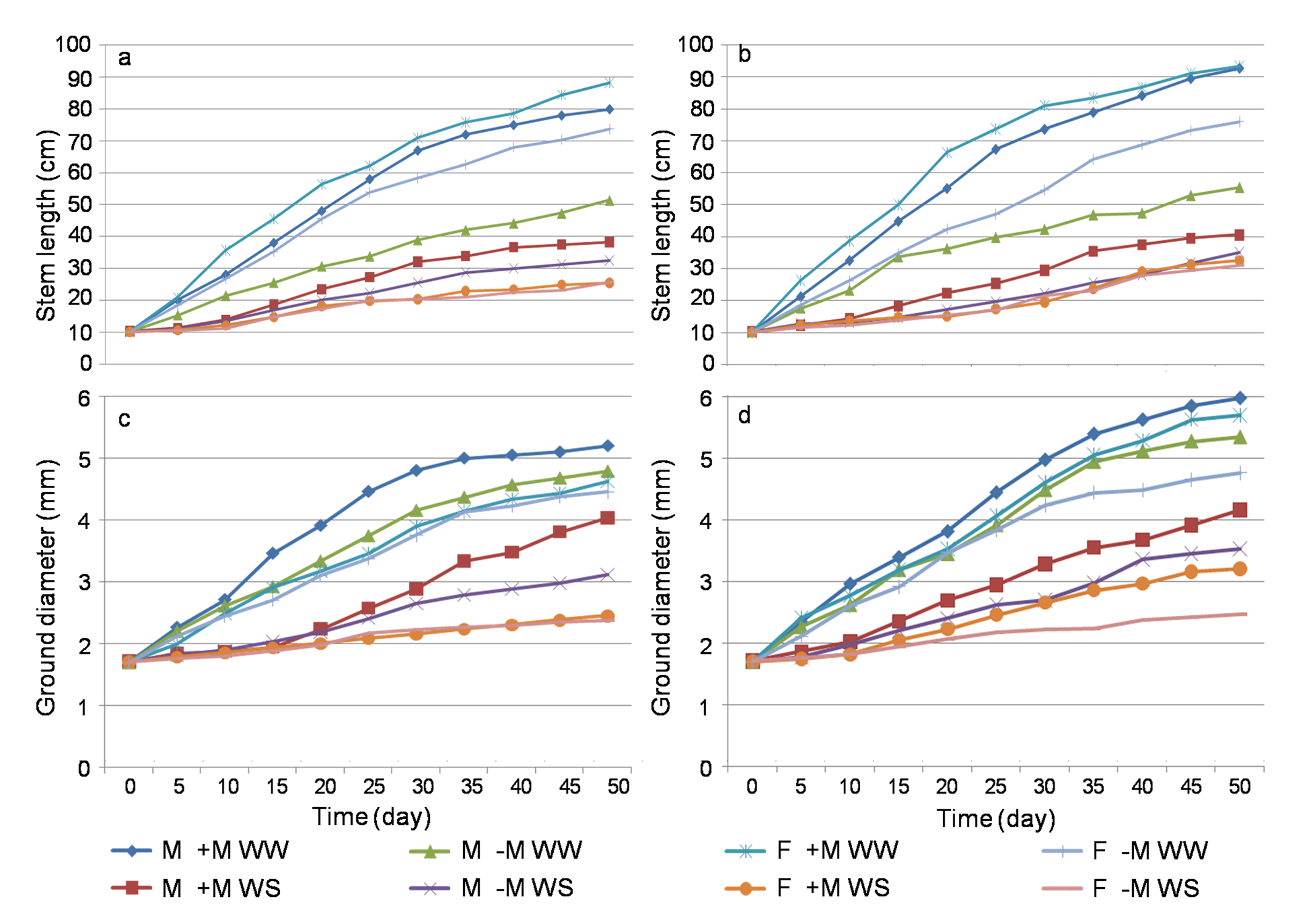

Supplement: Supplementary file 5 — Supplementary file5 (TIF 807 kb) [file 41598_2020_68112_MOESM5_ESM.tif]

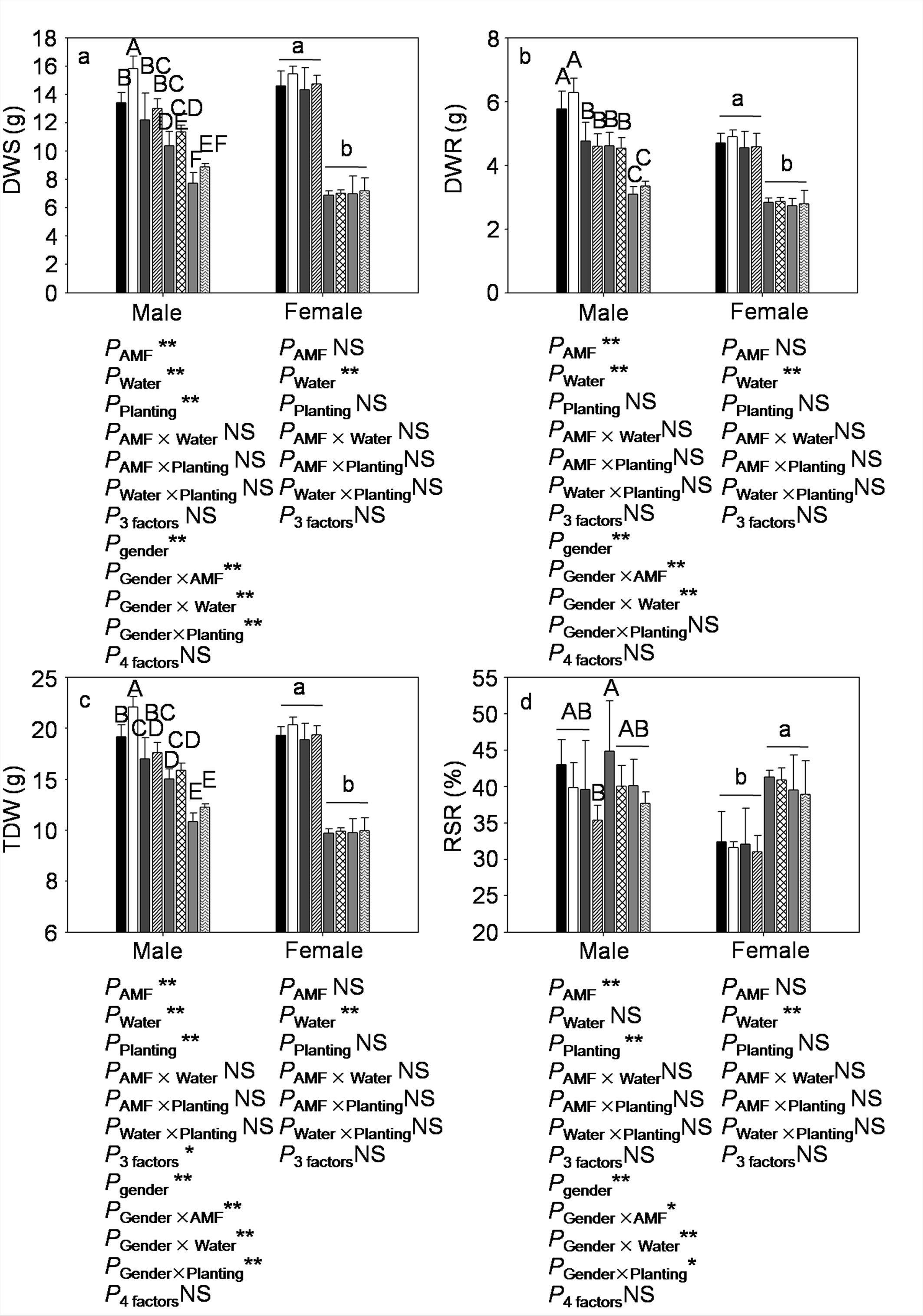

Supplement: Supplementary file 6 — Supplementary file6 (TIF 2234 kb) [file 41598_2020_68112_MOESM6_ESM.tif]

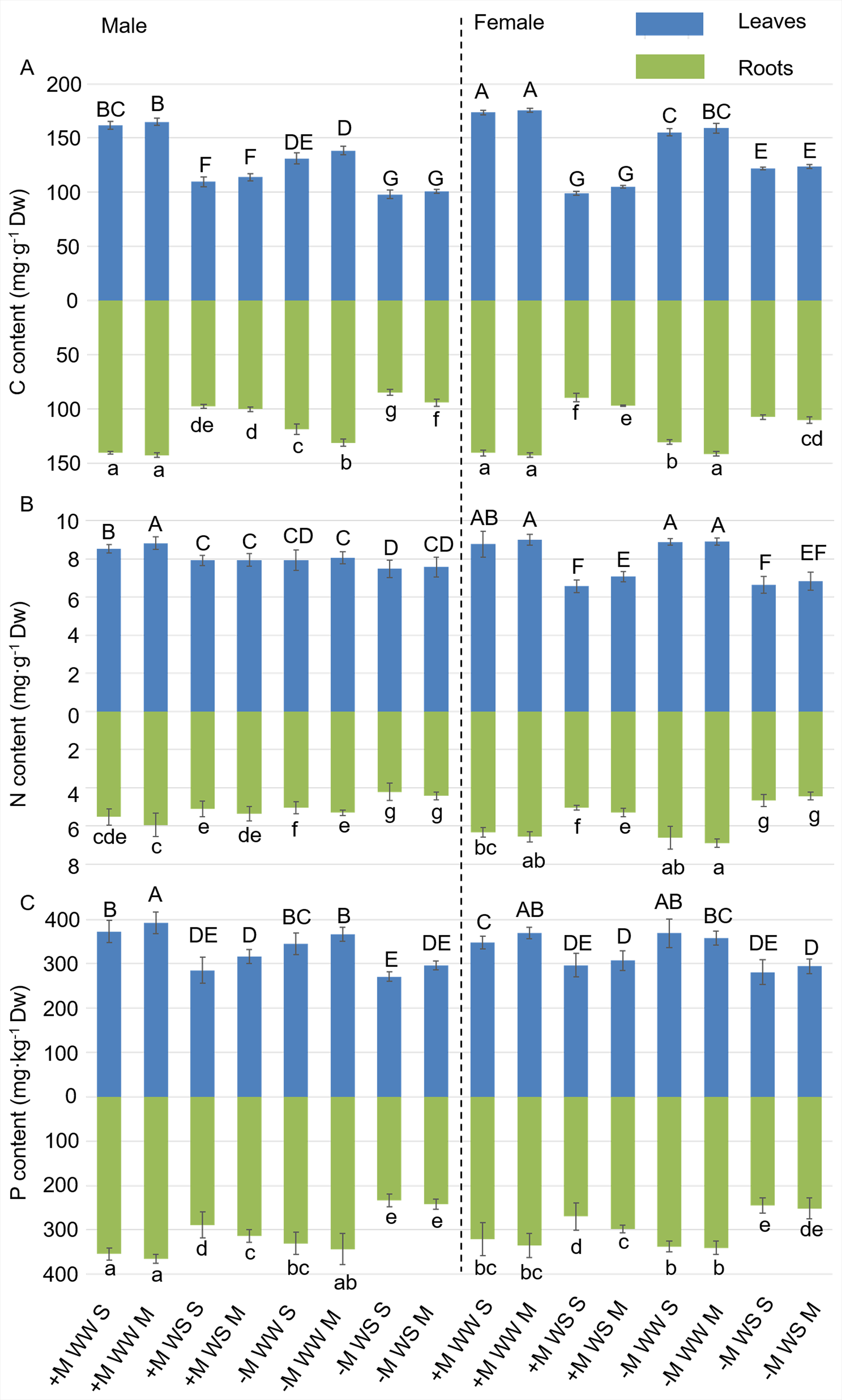

Supplement: Supplementary file 7 — Supplementary file7 (TIF 1660 kb) [file 41598_2020_68112_MOESM7_ESM.tif]

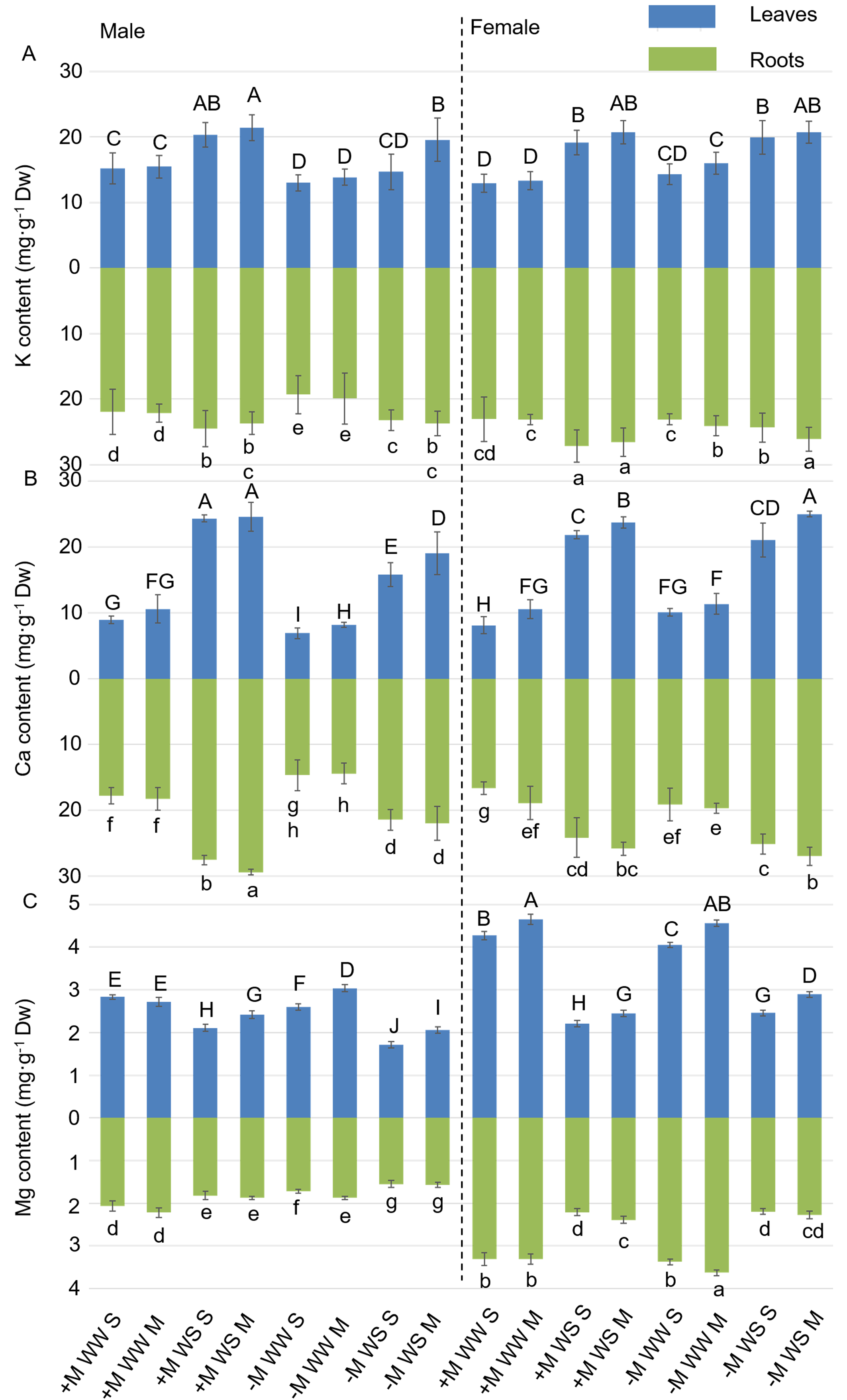

Supplement: Supplementary file 8 — Supplementary file8 (TIF 1125 kb) [file 41598_2020_68112_MOESM8_ESM.tif]
