# Supplementary material for: Gender-related responses of dioecious plant Populus cathayana to AMF, drought and planting pattern
Source: Sci Rep. 2020 Jul 13;10:11530. doi: 10.1038/s41598-020-68112-0 (PMC7359309; doi:10.1038/s41598-020-68112-0)
Supplement: Supplementary file 9 — Supplementary file9 (DOCX 45 kb) [file 41598_2020_68112_MOESM9_ESM.docx]

**Supplementary Table 1.** ANOVA results of elements

|  | | Leaf | | | | | | Root | | | | | |
| --- | --- | --- | --- | --- | --- | --- | --- | --- | --- | --- | --- | --- | --- |
|  |  | C | N | P | K | Ca | Mg | C | N | P | K | Ca | Mg |
| Male | *P*_AMF_ | ** | ** | ** | * | ** | ** | ** | ** | ** | ** | ** | ** |
|  | *P*_Water_ | ** | ** | ** | ** | ** | ** | ** | ** | ** | ** | ** | ** |
|  | *P*_Planting_ | NS | ** | ** | ** | ** | ** | ** | ** | ** | NS | ** | * |
|  | *P*_AMF×Water_ | ** | ** | NS | ** | ** | ** | * | ** | ** | ** | ** | NS |
|  | *P*_AMF×Planting_ | NS | NS | NS | ** | ** | * | * | NS | NS | NS | * | NS |
|  | *P*_Water×Planting_ | NS | NS | NS | ** | NS | NS | NS | NS | NS | NS | ** | NS |
|  | *P*_3 factors_ | NS | NS | NS | ** | ** | * | NS | NS | NS | NS | NS | NS |
| Female | *P*_AMF_ | NS | NS | NS | ** | ** | NS | ** | ** | ** | * | ** | NS |
|  | *P*_Water_ | ** | * | ** | ** | ** | ** | ** | ** | ** | * | ** | ** |
|  | *P*_Planting_ | * | * | ** | ** | ** | * | ** | ** | ** | * | ** | * |
|  | *P*_AMF×Water_ | ** | NS | ** | ** | ** | ** | ** | ** | ** | ** | NS | * |
|  | *P*_AMF×Planting_ | NS | * | * | NS | NS | NS | NS | * | * | ** | NS | NS |
|  | *P*_Water×Planting_ | NS | * | NS | NS | ** | NS | NS | ** | NS | NS | NS | NS |
|  | *P*_3 factors_ | NS | NS | ** | * | ** | NS | * | ** | NS | NS | * | NS |
| *P*_Gender_ | | ** | * | NS | * | ** | ** | ** | ** | ** | ** | ** | ** |
| *P*_AMF×Gender_ | | ** | * | ** | ** | ** | ** | ** | ** | ** | ** | ** | ** |
| *P*_Water×Gender_ | | ** | * | ** | ** | ** | ** | ** | ** | ** | ** | * | ** |
| *P*_Planting×Gender_ | | * | * | ** | ** | ** | ** | ** | ** | ** | NS | ** | * |
| *P*_AMF×Gender×Water_ | | ** | * | ** | ** | ** | ** | ** | ** | ** | ** | ** | * |
| *P*_AMF×Gender×Planting_ | | NS | * | * | ** | * | ** | NS | * | * | * | * | NS |
| *P*_Water×Gender×Planting_ | | NS | * | NS | * | * | NS | NS | * | NS | NS | * | NS |
| *P*_4 factors_ | | NS | NS | * | ** | ** | * | NS | * | NS | NS | NS | S |

Note: *: significant effect at 0.01≤ P ≤ 0.05; **: significant effect at P ≤ 0.01; NS: no significant effect.
