# Supplementary material for: Gender-related responses of dioecious plant Populus cathayana to AMF, drought and planting pattern
Source: Sci Rep. 2020 Jul 13;10:11530. doi: 10.1038/s41598-020-68112-0 (PMC7359309; doi:10.1038/s41598-020-68112-0)
Supplement: Supplementary file 10 — Supplementary file10 (DOCX 24 kb) [file 41598_2020_68112_MOESM10_ESM.docx]

**Supplementary Table 2.** Key contributions of factors in PCA

| Male | | | Female | | |
| --- | --- | --- | --- | --- | --- |
| Factors | PC1 | PC2 | Factors | PC1 | PC2 |
| DWS | 0.939 | 0.094 | DWS | 0.980 | -0.047 |
| TDW | 0.948 | 0.163 | DWR | 0.950 | -0.038 |
| Stem length | 0.928 | 0.072 | TDW | 0.983 | -0.045 |
| Ground diameter | 0.944 | -0.026 | Stem length | 0.984 | 0.010 |
| C content of leaves | 0.941 | -0.009 | Ground diameter | 0.958 | 0.133 |
| C content of roots | 0.955 | -0.088 | N content of leaves | 0.984 | 0.040 |
| P content of leaves | 0.964 | -0.114 | N content of roots | 0.951 | 0.044 |
| P content of roots | 0.956 | 0.068 | P content of leaves | 0.951 | 0.074 |
|  |  |  | Mg content of leaves | 0.961 | 0.079 |
